# Crystal structure of (*Z*)-3-{2-[(*Z*)-11*H*-indeno[1,2-*b*]quinoxalin-11-yl­idene]hydrazin­yl}-*N*-phenyl­but-2-enamide monohydrate

**DOI:** 10.1107/S2056989026002343

**Published:** 2026-03-11

**Authors:** Ghada A. Eldeken, Fatma A. El-Samahy, Ehab M. Zayed, Fayez H. Osman, Khaled Mahmoud, Galal H. Elgemeie, Peter G. Jones

**Affiliations:** a Department of Green Chemistry, Chemical Industries Research Institute, National Research Centre, 33 El-Buhouth St., Dokki, Giza, PO 12622, Egypt; b Pharmacognosy Department, National Research Centre, 33 El-Buhouth St., Dokki, Giza, PO 12622, Egypt; c Chemistry Department, Faculty of Science, Capital University, Helwan, Egypt; d Institut für Anorganische und Analytische Chemie, Technische Universität Braunschweig, Hagenring 30, D-38106 Braunschweig, Germany; Universität Greifswald, Germany

**Keywords:** crystal structure, hydrazin­yl, quinoxaline, hydrogen bonds

## Abstract

In the title compound, (*Z*)-3-{2-[(*Z*)-11*H*-indeno­[1,2-*b*]quinoxalin-11-yl­idene]hydrazin­yl}-*N*-phenyl­but-2-enamide monohydrate, C_25_H_19_N_5_O·H_2_O, the configurations around the C=N and C=C double bonds adjacent to the hydrazinyl moiety are both *Z*. Except for the phenyl group, the mol­ecule is almost planar, promoted by the three-centre intra­molecular N_hydrazin­yl_—H⋯(O_carbon­yl_, N_quinoxaline_) hydrogen bond. The water mol­ecule participates in three hydrogen bonds, as donor towards O_carbon­yl_ (within the asymmetric unit) and the other N_quinoxaline_ (*via* an inversion operator) and as acceptor from the amide N—H group (*via* a *b*-glide operator). The hydrogen bonds combine to form a layer structure parallel to the *ab* plane.

## Abstract 2

The title compound (*Z*)-3-{2-[(*Z*)-11*H*-indeno­[1,2-*b*]quinoxalin-11-yl­idene]hydrazin­yl}-*N*-phenyl­but-2-enamide monohydrate was synthesized and investigated using SCXRD. Except for the phenyl group, the mol­ecule is almost planar, and includes an intra­molecular N_hydrazin­yl_—H⋯(O_carbon­yl_, N_quinoxaline_) hydrogen-bond system. Hydrogen bonds combine to form a layer structure.

## 1. Chemical context

The pharmaceutical industry has shown great inter­est in quinoxaline derivatives because they display a wide spectrum of biological properties and can be used against various pathogens and diseases, *e.g.* bacteria, fungi, viruses, leishmania, tuberculosis, malaria or cancer (Deepika *et al.*, 2011 ▸; Pereira *et al.*, 2015 ▸).

Indeno­quinoxaline and its derivatives are an important class of nitro­gen-containing heterocycles and are useful inter­mediates in organic synthesis. They have furthermore been found to have applications in various therapies (Tseng *et al.*, 2016 ▸), as organic semiconductors (Sehlstedt *et al.*, 1998 ▸; Cheng *et al.*, 2011 ▸), anti­viral agents (Selvam *et al.*, 2013 ▸), α-glucosidase inhibitors (Khan *et al.*, 2014 ▸; Hameed *et al.*, 2024 ▸), anti-inflammatory agents (Schepetkin *et al.*, 2019 ▸), anti­microbial agents (Kotharkar & Shinde, 2006 ▸; Sawant *et al.*, 2025 ▸), acetyl­cholinesterase (AChE) inhibitors (Akondi *et al.*, 2017 ▸), anti­tumor agents (Tseng *et al.*, 2016 ▸; Saravana Mani *et al.*, 2018 ▸) or c-Jun N-terminal kinase (JNK) inhibitors (Schepetkin *et al.*, 2012 ▸) and as acid corrosion inhibitors for mild steel surfaces (Obot & Obi-Egbedi, 2010 ▸).

Acetoacetanilide is widely utilized in the synthesis of several heterocyclic compounds. Because of its reactivity and structural flexibility, it is a desirable building block for creating bioactive compounds (Singh *et al.*, 2019 ▸). The presence of an active methyl­ene group next to a carbonyl and an amide moiety renders it extremely reactive towards primary amines, forming Schiff bases [distinguished by the presence of an imine (—C=N—) functional group] by condensation reactions. These Schiff bases have a wide range of biological functions; they have shown encouraging anti­microbial (Raman *et al.*, 2001 ▸), anti­cancer (Subin Kumar, 2021 ▸) and anti­fungal (Deepa & Aravindakshan, 2004 ▸) properties. Imine-containing heterocyclic compounds display a varied chemical reactivity and often show considerable pharmacological effects, which have been attributed to the polarized C=N group (Kovrizhina *et al.*, 2021 ▸). Notable representatives of these compounds are azines, classified as hydrazine derivatives with the general formula *RR*′C=N—N=C*R′′R′′′*.

**Figure 1:**
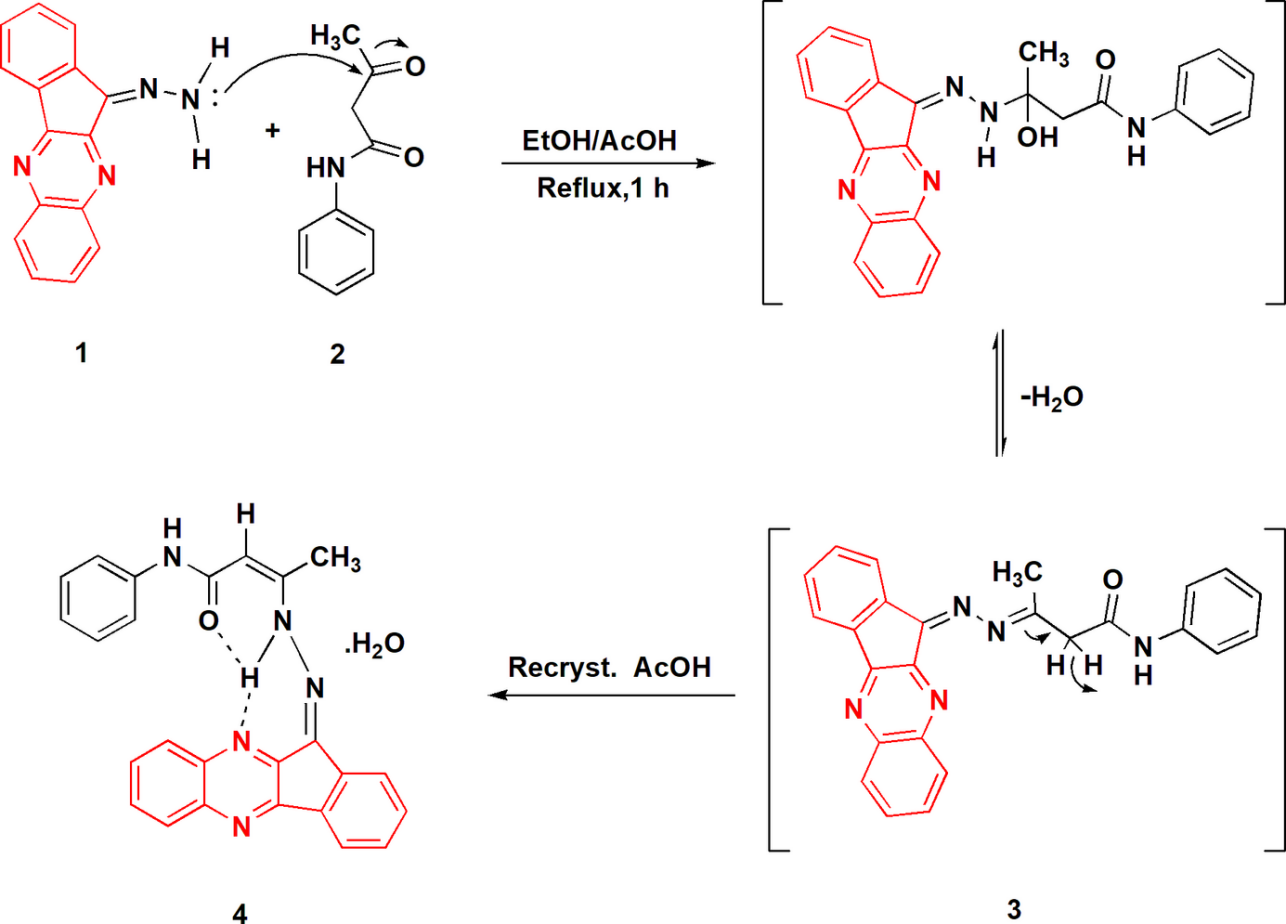
The synthesis of compound **4**.

Continuing our work on the indeno­[1,2-*b*]quinoxaline moiety (Eldeken *et al.*, 2022 ▸; El-Samahy *et al.*, 2023 ▸), we are attempting to synthesize new derivatives as potentially active compounds and to study their biological activities as anti­cancer agents. Treatment (Fig. 1 ▸) of 11-hydrazineyl­idene-11*H*-indeno­[1,2-*b*]quinoxaline (**1**) with the acetoacetanilide analogue 3-oxo-*N*-phenyl­butanamide (**2**) in ethanol in the presence of acetic acid led to the formation of (*Z*)-3-{2-[(*Z*)-11*H*-indeno­[1,2-*b*]quinoxalin-11-yl­idene]hydrazin­yl}-*N*-phen­yl­but-2-enamide monohydrate (**4**). Compound **4** was formed in good yield *via* a two-step mechanism involving nucleophilic addition of the primary amine to the carbonyl carbon to give the carbinolamine, followed by dehydration to yield the dienehydrazine **3**, containing an (=N—N=) group, followed by tautomerisation to form the mono-ene-hydrazine **4**, containing the (—NH—N=) group. The product was recrystallized from acetic acid.

The ^1^H NMR spectrum of **4** revealed the presence of two singlets at *δ* 2.33 and 5.27 ppm corresponding to =C—CH_3_ and =CH, respectively. The aromatic protons were observed as a multiplet at δ 7.35–8.06 (13 ArH) ppm and two singlets at *δ* 9.88, 15.05 ppm, attributed to OH and NH. The ^13^C NMR spectrum of **4** showed signals at *δ* 18.9 (CH_3_) and at 96.1 (=CH), beside the Ar—C signals.

In order to establish the chemical structure of the product **4**, its crystal structure was determined and is reported here. The structure was found to be a monohydrate; the water of crystallization probably arose both from the condensation step and also from the acetic acid used for the reaction and the recrystallization, but this was not investigated further. Henceforth, the compound number **4** refers to the monohydrate.
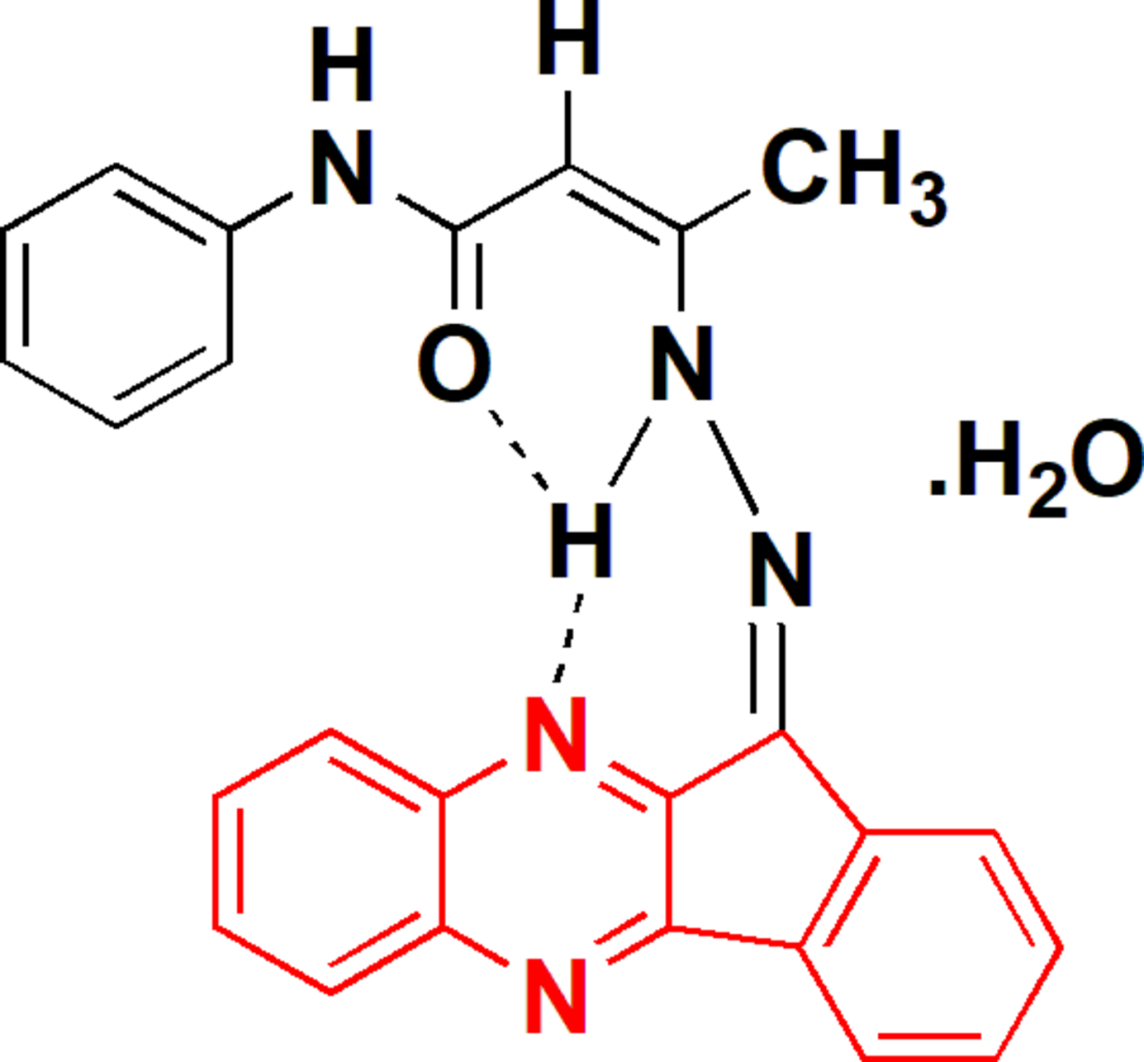


## 2. Structural commentary

**Figure 2:**
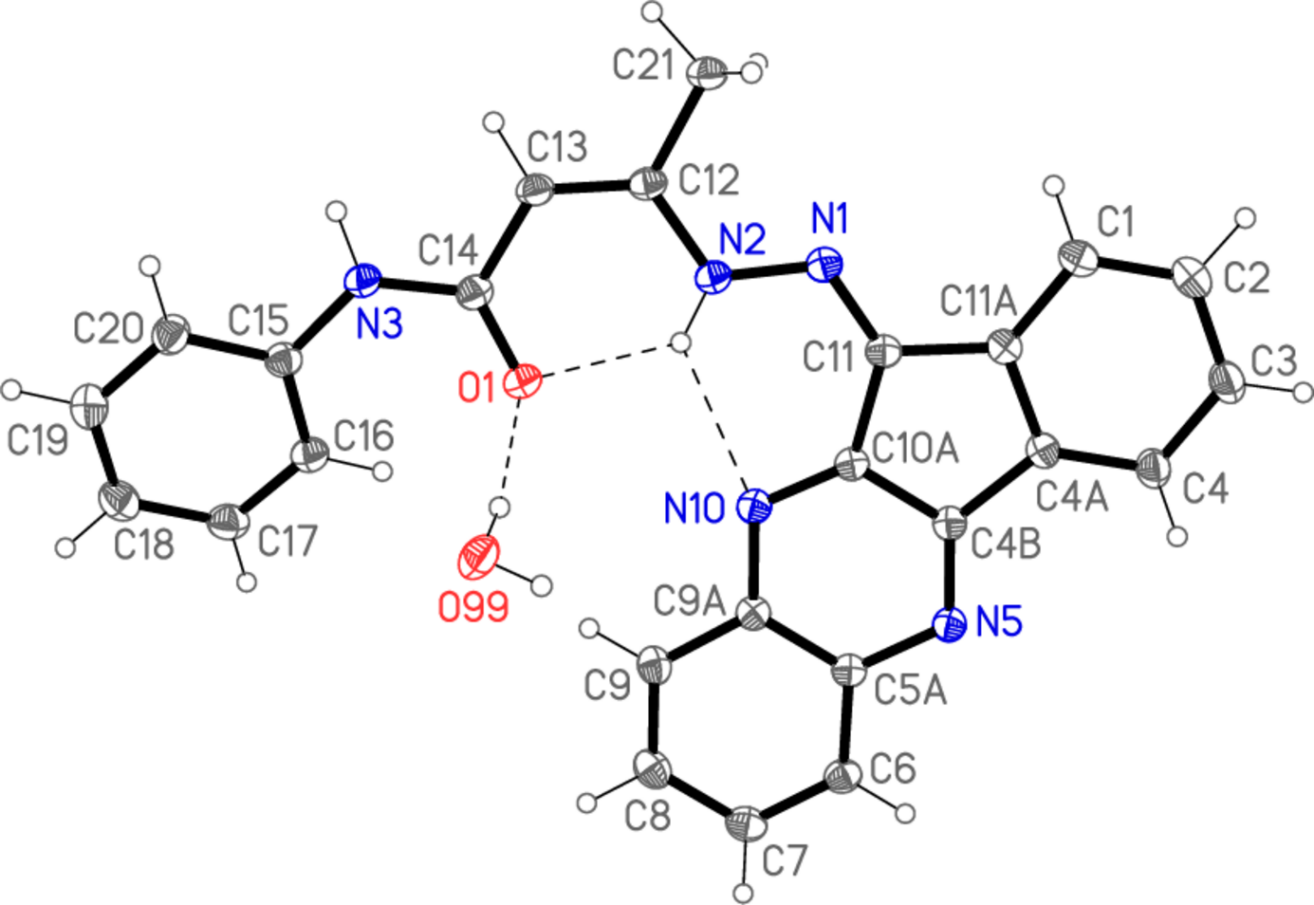
The asymmetric unit of compound **4** in the crystal. Dashed lines indicate hydrogen bonds. Ellipsoids correspond to 50% probability levels.

**Figure 3:**
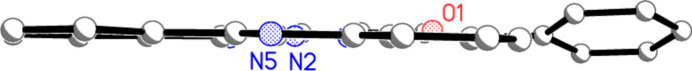
Side view of compound **4** (water mol­ecule and H atoms omitted).

The structure of compound **4** is shown in Fig. 2 ▸, with selected mol­ecular dimensions in Table 1 ▸. The configurations around the double bonds C11=N1 and C12=C13 are both *Z*. Except for the phenyl group, the entire mol­ecule is almost planar (Fig. 3 ▸), with an r.m.s. deviation of 0.04 Å for non-hydrogen atoms; the atom sequence C15–N3–C14–C13–C12–N2–N1–C11, which connects the two ring systems, is synperiplanar about the bond C12=C13 and anti­periplanar elsewhere. The phenyl group makes an angle of 20.31 (3)° with the main plane. The planarity is associated with the three-centre intra­molecular hydrogen bond N2—H02⋯(O1, N10), and the short intra­molecular contact H16⋯O1 might also be regarded as a ‘weak’ hydrogen bond (Table 2 ▸). Other hydrogen bonds are discussed in *Supra­molecular features*. Bond lengths and angles in and around the hydrazide group correspond reasonably well with the formal bond orders (see *Database survey*); some delocalization of multiple bonding would be expected, and the coordination at N2 is planar [it lies only 0.021 (8) Å out of the plane of its substituents H02, N1 and C12]. The fusing of five- and six-membered rings leads to the usual widening of the corresponding exocyclic bond angles, which are all > 128° and thus appreciably greater than the standard value for *sp*^2^ carbon atoms. In the five-membered ring, the angle at C11 [106.32 (9)°] is narrow, whereas N1—C11—C10*A* [131.15 (10)°] is extremely wide and the formal single bonds at C11 are, at *ca*. 1.47 Å, indeed appreciably longer than the other bonds.

We have recently published the related hydrazide structure (*E*)-2-(benzo[*d*]thia­zol-2-yl)-*N*′-[1-(4-bromo­phen­yl)ethyl­id­ene]acetohydrazide (Elboshi *et al.*, 2026 ▸), which also contains the atom sequence C(*sp*^2^)—NH—N=C, but with a C=O rather than a C=C double bond at the first atom. The bond lengths, in this order, are 1.3543 (15), 1.3764 (15) and 1.2942 (15) Å, compared to 1.3792 (14), 1.3458 (13) and 1.3004 (13) Å in **4** (see also *Database survey*).

## 3. Supra­molecular features

**Figure 4:**
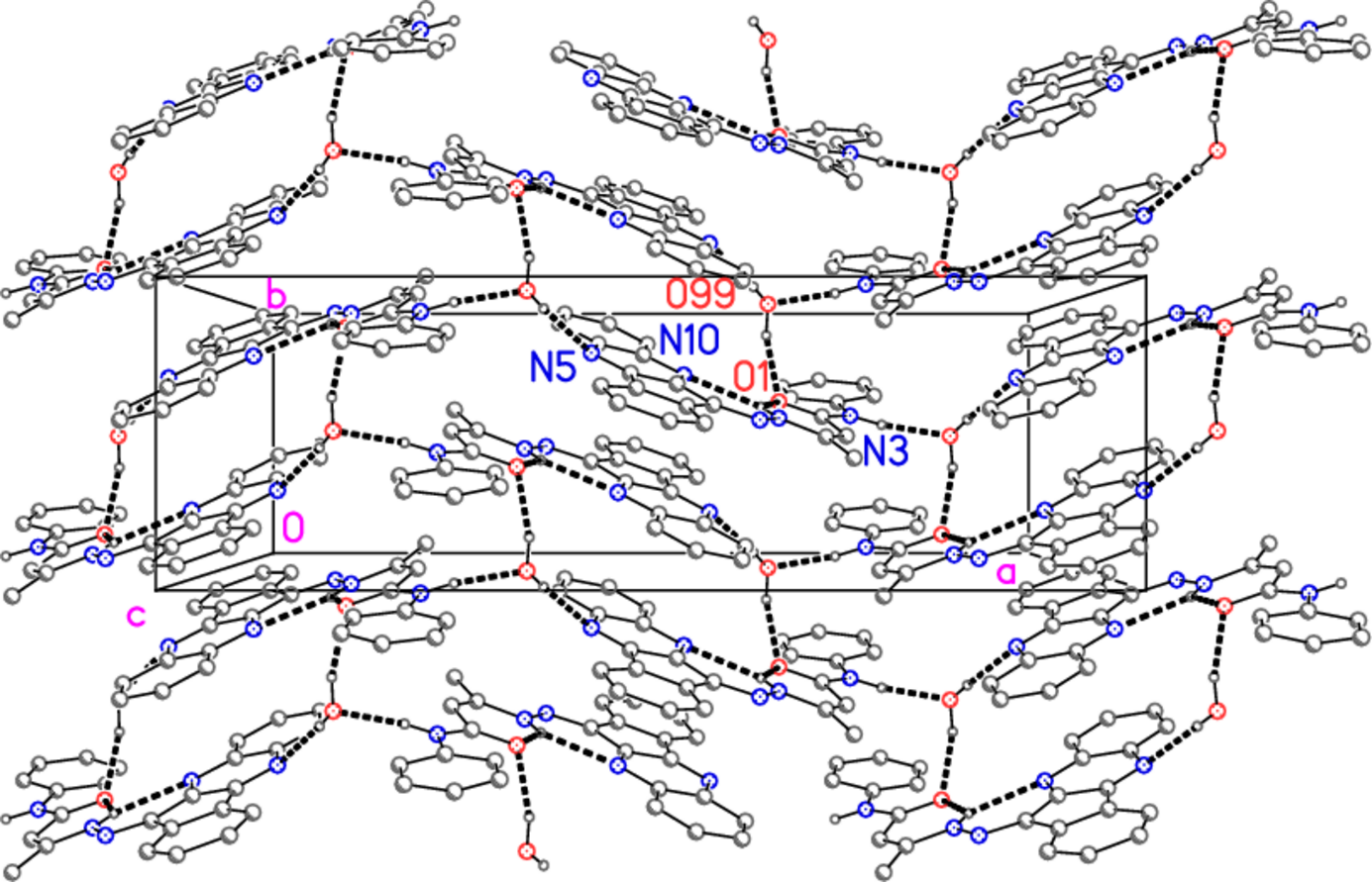
Packing of compound **4** showing the formation of a layer structure parallel to the *ab* plane. The view direction is parallel to the *c* axis in the region *z* ≃ 0.5. Thick dashed lines indicate classical hydrogen bonds. Hydrogen atoms not involved in hydrogen bonding are omitted. Atom labels correspond to the asymmetric unit.

The water mol­ecule participates in three hydrogen bonds, as donor towards O1 (within the asymmetric unit, Fig. 2 ▸) and N5 (*via* inversion), and as acceptor from the amide group N3—H (*via* a *b* glide plane). The classical hydrogen bonds (Table 2 ▸) combine to form a layer structure parallel to the *ab* plane (Fig. 4 ▸).

## 4. Database survey

Searches were conducted using CSD Version 6.00 (update August 2025; Groom *et al.*, 2016 ▸) and the ConQuest routine (Bruno *et al.*, 2002 ▸), Version 2025.2.0.

A search for the substituted hydrazine moiety (C,C)–C^3^–N^3^(H)–N^2^–C^3^–(C,C) was conducted, where the superscripts refer to coordination numbers. Disordered structures and those involving metals were excluded; C—C and C—N bond types were restricted to ‘acyclic’, but no explicit restrictions were placed on bond orders. This led to 1244 hits. The 1472 values for the N—N bond length gave a mean value of 1.340 (36) Å, corresponding well to the value of 1.3458 (13) Å in **4**; similarly, the 1472 values for the N^2^=C^3^ bond length gave a mean value of 1.305 (21) Å, *cf*. 1.3004 (13) Å in **4**.

Extending the search fragment to phenyl-NH–C(=O)–C–C^3^–N^3^(H)–N^2^–C^3^–(C,C), as in **4**, gave one hit, namely 2-[2-(2,6-dioxo­cyclo­hexyl­idene)hydrazinyl-*N*-phenyl­benzamide] chloro­form solvate (refcode GUCBAL; Bao *et al.*, 2024 ▸), in which, however, the central C—C^3^ bond forms part of a phenyl ring.

## 5. Synthesis and crystallization

A mixture of 11-hydrazineyl­idene-11*H*-indeno­[1,2-*b*]quinoxaline **1** (0.01 mol) and 3-oxo-*N*-phenyl­butanamide **2** (0.01 mol) in ethanol (20 ml) and acetic acid (10 ml) was refluxed for 1 h at 353 K. After completion of the reaction (TLC), the solid precipitate thus formed was filtered off and recrystallized from acetic acid. Orange solid; yield 90%; m.p. 501 K; IR (KBr, cm^−1^): *ν* 3551, 3055, 1627 (C=N) cm^−1^; ^1^H NMR (500 MHz, DMSO-*d_6_*): *δ* 2.33 (*s*, 3 H, =C—CH_3_), 5.27 (=H), 7.35–8.06 (*m*, 13 ArH), 9.88, 15.05 (2*s*, OH, NH) ppm; ^13^C NMR (125 MHz, DMSO-*d_6_*): δ 18.9 (=C—CH_3_), 96.1 (=CH), 119.3, 120.8, 122.6, 123.1, 129.3, 129.7, 129.9, 130.1, 130.9, 132.3, 134.3, 135.4, 141.2, 153.4 and 166.8 ppm; ESI-MS *m*/*z* (%) 406 (*M*^+^ + 1, 100%); Analysis calculated for C_25_H_19_N_5_O (405.46): C 74.06, H 4.72, N 17.27; found C 74.12, H 4.79, N 17.19%.

## 6. Refinement

Details of data collection and structure refinement are summarized in Table 3 ▸. The hydrogen atoms of the NH groups and the water mol­ecule were refined freely. The methyl group was refined as an idealized rigid group with C—H = 0.98 Å, H—C—H = 109.5°, allowed to rotate but not tip (AFIX 137). Other hydrogen atoms were included using a riding model starting from calculated positions with C(*sp*^2^)—H = 0.95 Å. The *U*_iso_(H) values were fixed at 1.5 × *U*_eq_ of the parent carbon atoms for the methyl group and 1.2 × *U*_eq_ for the other C-bound hydrogen atoms.

## Supplementary Material

Crystal structure: contains datablock(s) I, global. DOI: 10.1107/S2056989026002343/yz2074sup1.cif

Structure factors: contains datablock(s) I. DOI: 10.1107/S2056989026002343/yz2074Isup2.hkl

Supporting information file. DOI: 10.1107/S2056989026002343/yz2074Isup3.cml

CCDC reference: 2535107

Additional supporting information:  crystallographic information; 3D view; checkCIF report

## Figures and Tables

**Table 1:** Selected geometric parameters (Å, °)

C10*A*—C11	1.4691 (15)	N2—C12	1.3792 (14)
C11*A*—C11	1.4678 (15)	N3—C14	1.3602 (15)
N1—C11	1.3004 (14)	C12—C13	1.3570 (15)
N1—N2	1.3458 (13)	C13—C14	1.4597 (15)
			
C4—C4*A*—C4*B*	130.44 (10)	N1—C11—C11*A*	122.53 (10)
N5—C4*B*—C4*A*	128.52 (10)	N1—C11—C10*A*	131.15 (10)
N10—C10*A*—C11	128.42 (10)	C11*A*—C11—C10*A*	106.32 (9)
C1—C11*A*—C11	130.32 (10)	C13—C12—N2	121.72 (10)
C11—N1—N2	117.74 (9)	C12—C13—C14	124.36 (10)
N1—N2—C12	118.30 (9)	N3—C14—C13	114.33 (10)
C14—N3—C15	128.53 (10)		
			
C11—N1—N2—C12	−176.41 (10)	C15—N3—C14—C13	176.42 (11)
N1—N2—C12—C13	−179.84 (10)	C12—C13—C14—N3	174.77 (11)
N2—C12—C13—C14	−0.31 (18)		

**Table 2:** Hydrogen-bond geometry (Å, °)

*D*—H⋯*A*	*D*—H	H⋯*A*	*D*⋯*A*	*D*—H⋯*A*
N2—H02⋯N10	0.905 (18)	2.251 (18)	2.9178 (13)	130.2 (14)
N2—H02⋯O1	0.905 (18)	2.008 (17)	2.6860 (13)	130.5 (15)
N3—H03⋯O99^i^	0.916 (18)	1.920 (19)	2.8305 (13)	172.7 (17)
O99—H99*B*⋯N5^ii^	0.88 (2)	2.04 (2)	2.9032 (14)	168 (2)
O99—H99*A*⋯O1	0.89 (3)	1.91 (3)	2.7802 (14)	166 (2)
C16—H16⋯O1	0.95	2.32	2.9050 (15)	119

Symmetry codes: (i) 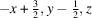; (ii) 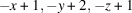.

**Table 3:** Experimental details

Crystal data	
Chemical formula	C_25_H_19_N_5_O·H_2_O
*M*_r_	423.47
Crystal system, space group	Orthorhombic, *Pbca*
Temperature (K)	100
*a*, *b*, *c* (Å)	23.0033 (6), 7.3011 (2), 24.3621 (6)
*V* (Å^3^)	4091.58 (18)
*Z*	8
Radiation type	Mo *K*α
μ (mm^−1^)	0.09
Crystal size (mm)	0.2 × 0.05 × 0.05

Data collection	
Diffractometer	XtaLAB Synergy
Absorption correction	Multi-scan (*CrysAlis PRO*; Rigaku OD, 2024 ▸)
*T*_min_, *T*_max_	0.766, 1.000
No. of measured, independent and observed [*I* > 2σ(*I*)] reflections	141178, 6816, 5392
*R*_int_	0.050
θ values (°)	θ_max_ = 31.5, θ_min_ = 2.4
(sin θ/λ)_max_ (Å^−1^)	0.735

Refinement	
*R*[*F*^2^ > 2σ(*F*^2^)], *wR*(*F*^2^), *S*	0.044, 0.129, 1.05
No. of reflections	6816
No. of parameters	306
H-atom treatment	H atoms treated by a mixture of independent and constrained refinement
Δρ_max_, Δρ_min_ (e Å^−3^)	0.45, −0.25

Computer programs: *CrysAlis PRO* (Rigaku OD, 2024 ▸), *SHELXT* (Sheldrick, 2015*a* ▸), *SHELXL2019/3* (Sheldrick, 2015*b* ▸) and *XP* (Bruker, 1998 ▸), *publCIF* (Westrip, 2010 ▸).

## supplementary crystallographic information

### (Z)-3-{2-[(Z)-11H-Indeno[1,2-b]quinoxalin-11-ylidene]hydrazinyl}-N-phenylbut-2-enamide monohydrate Crystal data

**Table:**

C_25_H_19_N_5_O·H_2_O	*D*_x_ = 1.375 Mg m^−^^3^
*M**_r_* = 423.47	Mo *K*α radiation, λ = 0.71073 Å
Orthorhombic, *P**b**c**a*	Cell parameters from 51739 reflections
*a* = 23.0033 (6) Å	θ = 2.4–34.3°
*b* = 7.3011 (2) Å	µ = 0.09 mm^−^^1^
*c* = 24.3621 (6) Å	*T* = 100 K
*V* = 4091.58 (18) Å^3^	Prism, orange
*Z* = 8	0.2 × 0.05 × 0.05 mm
*F*(000) = 1776	

### (Z)-3-{2-[(Z)-11H-Indeno[1,2-b]quinoxalin-11-ylidene]hydrazinyl}-N-phenylbut-2-enamide monohydrate Data collection

**Table:**

XtaLAB Synergy diffractometer	6816 independent reflections
Radiation source: micro-focus sealed X-ray tube, PhotonJet (Mo) X-ray Source	5392 reflections with *I* > 2σ(*I*)
Mirror monochromator	*R*_int_ = 0.050
Detector resolution: 10.0000 pixels mm^-1^	θ_max_ = 31.5°, θ_min_ = 2.4°
ω scans	*h* = −33→33
Absorption correction: multi-scan (CrysAlisPro; Rigaku OD, 2024)	*k* = −10→10
*T*_min_ = 0.766, *T*_max_ = 1.000	*l* = −35→35
141178 measured reflections	

### (Z)-3-{2-[(Z)-11H-Indeno[1,2-b]quinoxalin-11-ylidene]hydrazinyl}-N-phenylbut-2-enamide monohydrate Refinement

**Table:**

Refinement on *F*^2^	Primary atom site location: dual
Least-squares matrix: full	Hydrogen site location: mixed
*R*[*F*^2^ > 2σ(*F*^2^)] = 0.044	H atoms treated by a mixture of independent and constrained refinement
*wR*(*F*^2^) = 0.129	*w* = 1/[σ^2^(*F*_o_^2^) + (0.0614*P*)^2^ + 2.0644*P*] where *P* = (*F*_o_^2^ + 2*F*_c_^2^)/3
*S* = 1.05	(Δ/σ)_max_ = 0.001
6816 reflections	Δρ_max_ = 0.45 e Å^−^^3^
306 parameters	Δρ_min_ = −0.25 e Å^−^^3^
0 restraints	

### (Z)-3-{2-[(Z)-11H-Indeno[1,2-b]quinoxalin-11-ylidene]hydrazinyl}-N-phenylbut-2-enamide monohydrate Special details

**Table:**

Refinement. Hydrogen aoms of the NH groups and the water molecule were refined freely.

### (Z)-3-{2-[(Z)-11H-Indeno[1,2-b]quinoxalin-11-ylidene]hydrazinyl}-N-phenylbut-2-enamide monohydrate Fractional atomic coordinates and isotropic or equivalent isotropic displacement parameters (Å^2^)

**Table:**

	*x*	*y*	*z*	*U*_iso_*/*U*_eq_	
C1	0.55926 (5)	0.53628 (16)	0.67892 (5)	0.0219 (2)	
H1	0.597628	0.491157	0.683959	0.026*	
C2	0.52021 (6)	0.54307 (18)	0.72255 (5)	0.0258 (2)	
H2	0.532377	0.503694	0.757899	0.031*	
C3	0.46343 (6)	0.60678 (19)	0.71526 (5)	0.0267 (2)	
H3	0.437545	0.608780	0.745636	0.032*	
C4	0.44426 (5)	0.66720 (17)	0.66434 (5)	0.0228 (2)	
H4	0.405626	0.710036	0.659337	0.027*	
C4A	0.48347 (5)	0.66295 (15)	0.62092 (4)	0.0179 (2)	
C4B	0.47645 (5)	0.71801 (15)	0.56372 (4)	0.01679 (19)	
N5	0.43117 (4)	0.79265 (13)	0.54008 (4)	0.01806 (18)	
C5A	0.43816 (5)	0.83076 (15)	0.48502 (4)	0.01676 (19)	
C6	0.39134 (5)	0.91033 (16)	0.45643 (5)	0.0203 (2)	
H6	0.355943	0.935279	0.475080	0.024*	
C7	0.39666 (5)	0.95207 (17)	0.40158 (5)	0.0224 (2)	
H7	0.364926	1.005411	0.382419	0.027*	
C8	0.44915 (5)	0.91578 (17)	0.37380 (5)	0.0226 (2)	
H8	0.452567	0.946315	0.336031	0.027*	
C9	0.49542 (5)	0.83703 (16)	0.40042 (5)	0.0204 (2)	
H9	0.530381	0.812397	0.381017	0.024*	
C9A	0.49099 (5)	0.79269 (15)	0.45668 (4)	0.01694 (19)	
N10	0.53842 (4)	0.71632 (13)	0.48283 (4)	0.01743 (18)	
C10A	0.53009 (5)	0.68124 (14)	0.53508 (4)	0.01624 (19)	
C11A	0.54060 (5)	0.59738 (15)	0.62769 (4)	0.0175 (2)	
N1	0.62469 (4)	0.54870 (13)	0.56921 (4)	0.01817 (18)	
N2	0.64825 (4)	0.55622 (14)	0.51869 (4)	0.01852 (18)	
H02	0.6285 (7)	0.598 (3)	0.4891 (7)	0.032 (4)*	
N3	0.74054 (4)	0.56738 (15)	0.36773 (4)	0.02049 (19)	
H03	0.7780 (8)	0.533 (3)	0.3750 (7)	0.036 (5)*	
O1	0.65281 (4)	0.61976 (13)	0.41018 (4)	0.02395 (18)	
C11	0.57135 (5)	0.60445 (15)	0.57494 (4)	0.01708 (19)	
C12	0.70363 (5)	0.48796 (15)	0.51146 (5)	0.0181 (2)	
C13	0.73033 (5)	0.49112 (16)	0.46180 (5)	0.0194 (2)	
H13	0.768566	0.442609	0.459439	0.023*	
C14	0.70420 (5)	0.56409 (16)	0.41176 (5)	0.0196 (2)	
C15	0.72818 (5)	0.62003 (16)	0.31326 (5)	0.0194 (2)	
C16	0.67195 (5)	0.63792 (17)	0.29244 (5)	0.0227 (2)	
H16	0.639271	0.619806	0.315658	0.027*	
C17	0.66414 (6)	0.68253 (18)	0.23735 (5)	0.0256 (2)	
H17	0.625836	0.692895	0.223104	0.031*	
C18	0.71115 (6)	0.71209 (18)	0.20294 (5)	0.0264 (2)	
H18	0.705214	0.744109	0.165558	0.032*	
C19	0.76714 (6)	0.69432 (18)	0.22378 (5)	0.0266 (2)	
H19	0.799672	0.714469	0.200533	0.032*	
C20	0.77573 (5)	0.64738 (18)	0.27830 (5)	0.0235 (2)	
H20	0.814132	0.633652	0.292055	0.028*	
C21	0.73298 (5)	0.41143 (16)	0.56135 (5)	0.0203 (2)	
H21A	0.737643	0.508452	0.588802	0.031*	
H21B	0.771258	0.363308	0.551179	0.031*	
H21C	0.709222	0.312495	0.576713	0.031*	
O99	0.64052 (4)	0.99027 (14)	0.38653 (4)	0.02674 (19)	
H99A	0.6381 (9)	0.871 (3)	0.3938 (9)	0.055 (6)*	
H99B	0.6224 (10)	1.052 (3)	0.4124 (9)	0.059 (6)*	

### (Z)-3-{2-[(Z)-11H-Indeno[1,2-b]quinoxalin-11-ylidene]hydrazinyl}-N-phenylbut-2-enamide monohydrate Atomic displacement parameters (Å^2^)

**Table:**

	*U*^11^	*U*^22^	*U*^33^	*U*^12^	*U*^13^	*U*^23^
C1	0.0225 (5)	0.0222 (5)	0.0211 (5)	−0.0005 (4)	−0.0039 (4)	0.0012 (4)
C2	0.0302 (6)	0.0282 (6)	0.0191 (5)	−0.0011 (5)	−0.0021 (4)	0.0035 (4)
C3	0.0277 (6)	0.0335 (6)	0.0189 (5)	−0.0001 (5)	0.0024 (4)	0.0036 (5)
C4	0.0219 (5)	0.0277 (6)	0.0188 (5)	−0.0007 (4)	0.0024 (4)	0.0018 (4)
C4A	0.0185 (5)	0.0180 (5)	0.0173 (4)	−0.0018 (4)	−0.0002 (4)	0.0003 (4)
C4B	0.0154 (4)	0.0172 (5)	0.0178 (4)	−0.0024 (4)	−0.0002 (4)	−0.0006 (4)
N5	0.0170 (4)	0.0194 (4)	0.0178 (4)	−0.0005 (3)	−0.0001 (3)	−0.0011 (3)
C5A	0.0162 (4)	0.0164 (4)	0.0177 (4)	−0.0017 (4)	−0.0019 (3)	−0.0012 (4)
C6	0.0170 (5)	0.0217 (5)	0.0222 (5)	0.0005 (4)	−0.0028 (4)	−0.0013 (4)
C7	0.0211 (5)	0.0235 (5)	0.0227 (5)	−0.0005 (4)	−0.0065 (4)	0.0000 (4)
C8	0.0240 (5)	0.0252 (6)	0.0185 (5)	−0.0042 (4)	−0.0042 (4)	0.0005 (4)
C9	0.0199 (5)	0.0236 (5)	0.0176 (5)	−0.0036 (4)	−0.0005 (4)	−0.0005 (4)
C9A	0.0153 (4)	0.0175 (5)	0.0180 (4)	−0.0027 (4)	−0.0022 (3)	−0.0012 (4)
N10	0.0156 (4)	0.0189 (4)	0.0178 (4)	−0.0016 (3)	−0.0012 (3)	−0.0015 (3)
C10A	0.0154 (4)	0.0156 (4)	0.0177 (5)	−0.0014 (4)	−0.0014 (3)	−0.0010 (3)
C11A	0.0187 (5)	0.0161 (5)	0.0177 (4)	−0.0015 (4)	−0.0010 (4)	−0.0001 (4)
N1	0.0172 (4)	0.0177 (4)	0.0196 (4)	−0.0006 (3)	−0.0006 (3)	−0.0002 (3)
N2	0.0151 (4)	0.0211 (4)	0.0193 (4)	0.0012 (3)	−0.0009 (3)	0.0003 (3)
N3	0.0152 (4)	0.0263 (5)	0.0199 (4)	0.0024 (4)	−0.0017 (3)	0.0006 (4)
O1	0.0178 (4)	0.0305 (4)	0.0235 (4)	0.0056 (3)	0.0002 (3)	0.0023 (3)
C11	0.0163 (4)	0.0165 (4)	0.0185 (4)	−0.0009 (4)	−0.0018 (3)	−0.0005 (4)
C12	0.0151 (4)	0.0176 (5)	0.0217 (5)	−0.0001 (4)	−0.0022 (4)	0.0001 (4)
C13	0.0155 (4)	0.0210 (5)	0.0218 (5)	0.0021 (4)	−0.0018 (4)	0.0010 (4)
C14	0.0174 (5)	0.0206 (5)	0.0209 (5)	0.0009 (4)	−0.0006 (4)	−0.0006 (4)
C15	0.0195 (5)	0.0196 (5)	0.0192 (5)	0.0008 (4)	−0.0015 (4)	−0.0011 (4)
C16	0.0190 (5)	0.0262 (6)	0.0230 (5)	0.0019 (4)	−0.0013 (4)	0.0006 (4)
C17	0.0243 (6)	0.0274 (6)	0.0251 (6)	0.0029 (5)	−0.0046 (4)	0.0008 (4)
C18	0.0327 (6)	0.0252 (6)	0.0214 (5)	0.0006 (5)	−0.0019 (5)	0.0005 (4)
C19	0.0274 (6)	0.0293 (6)	0.0231 (5)	−0.0014 (5)	0.0038 (4)	−0.0004 (5)
C20	0.0195 (5)	0.0278 (6)	0.0234 (5)	−0.0006 (4)	0.0005 (4)	−0.0008 (4)
C21	0.0168 (5)	0.0212 (5)	0.0230 (5)	0.0012 (4)	−0.0033 (4)	0.0023 (4)
O99	0.0195 (4)	0.0281 (5)	0.0326 (5)	0.0016 (3)	0.0068 (3)	−0.0011 (4)

### (Z)-3-{2-[(Z)-11H-Indeno[1,2-b]quinoxalin-11-ylidene]hydrazinyl}-N-phenylbut-2-enamide monohydrate Geometric parameters (Å, º)

**Table:**

C1—C2	1.3925 (17)	C13—C14	1.4597 (15)
C1—C11A	1.3932 (15)	C15—C16	1.3953 (16)
C2—C3	1.3978 (18)	C15—C20	1.4007 (16)
C3—C4	1.3885 (16)	C16—C17	1.3927 (17)
C4—C4A	1.3905 (16)	C17—C18	1.3853 (18)
C4A—C11A	1.4084 (15)	C18—C19	1.3905 (19)
C4A—C4B	1.4593 (15)	C19—C20	1.3859 (17)
C4B—N5	1.3091 (14)	C1—H1	0.9500
C4B—C10A	1.4428 (15)	C2—H2	0.9500
N5—C5A	1.3793 (14)	C3—H3	0.9500
C5A—C6	1.4081 (15)	C4—H4	0.9500
C5A—C9A	1.4251 (15)	C6—H6	0.9500
C6—C7	1.3761 (16)	C7—H7	0.9500
C7—C8	1.4093 (17)	C8—H8	0.9500
C8—C9	1.3725 (16)	C9—H9	0.9500
C9—C9A	1.4120 (15)	N2—H02	0.905 (18)
C9A—N10	1.3809 (14)	N3—H03	0.916 (18)
N10—C10A	1.3125 (14)	C13—H13	0.9500
C10A—C11	1.4691 (15)	C16—H16	0.9500
C11A—C11	1.4678 (15)	C17—H17	0.9500
N1—C11	1.3004 (14)	C18—H18	0.9500
N1—N2	1.3458 (13)	C19—H19	0.9500
N2—C12	1.3792 (14)	C20—H20	0.9500
N3—C14	1.3602 (15)	C21—H21A	0.9800
N3—C15	1.4106 (14)	C21—H21B	0.9800
O1—C14	1.2507 (14)	C21—H21C	0.9800
C12—C13	1.3570 (15)	O99—H99A	0.89 (3)
C12—C21	1.4985 (15)	O99—H99B	0.88 (2)
			
C2—C1—C11A	118.27 (11)	C17—C16—C15	119.46 (11)
C1—C2—C3	121.17 (11)	C18—C17—C16	121.26 (12)
C4—C3—C2	121.07 (11)	C17—C18—C19	119.17 (12)
C3—C4—C4A	117.81 (11)	C20—C19—C18	120.35 (12)
C4—C4A—C11A	121.58 (10)	C19—C20—C15	120.43 (11)
C4—C4A—C4B	130.44 (10)	C2—C1—H1	120.9
C11A—C4A—C4B	107.98 (9)	C11A—C1—H1	120.9
N5—C4B—C10A	123.03 (10)	C1—C2—H2	119.4
N5—C4B—C4A	128.52 (10)	C3—C2—H2	119.4
C10A—C4B—C4A	108.42 (9)	C4—C3—H3	119.5
C4B—N5—C5A	114.77 (9)	C2—C3—H3	119.5
N5—C5A—C6	118.36 (10)	C3—C4—H4	121.1
N5—C5A—C9A	122.09 (10)	C4A—C4—H4	121.1
C6—C5A—C9A	119.54 (10)	C7—C6—H6	119.9
C7—C6—C5A	120.24 (11)	C5A—C6—H6	119.9
C6—C7—C8	120.06 (11)	C6—C7—H7	120.0
C9—C8—C7	121.08 (11)	C8—C7—H7	120.0
C8—C9—C9A	119.91 (11)	C9—C8—H8	119.5
N10—C9A—C9	118.90 (10)	C7—C8—H8	119.5
N10—C9A—C5A	121.93 (10)	C8—C9—H9	120.0
C9—C9A—C5A	119.16 (10)	C9A—C9—H9	120.0
C10A—N10—C9A	114.26 (9)	N1—N2—H02	122.7 (11)
N10—C10A—C4B	123.89 (10)	C12—N2—H02	118.9 (11)
N10—C10A—C11	128.42 (10)	C14—N3—H03	114.9 (11)
C4B—C10A—C11	107.68 (9)	C15—N3—H03	116.6 (12)
C1—C11A—C4A	120.08 (10)	C12—C13—H13	117.8
C1—C11A—C11	130.32 (10)	C14—C13—H13	117.8
C4A—C11A—C11	109.58 (9)	C17—C16—H16	120.3
C11—N1—N2	117.74 (9)	C15—C16—H16	120.3
N1—N2—C12	118.30 (9)	C18—C17—H17	119.4
C14—N3—C15	128.53 (10)	C16—C17—H17	119.4
N1—C11—C11A	122.53 (10)	C17—C18—H18	120.4
N1—C11—C10A	131.15 (10)	C19—C18—H18	120.4
C11A—C11—C10A	106.32 (9)	C20—C19—H19	119.8
C13—C12—N2	121.72 (10)	C18—C19—H19	119.8
C13—C12—C21	121.71 (10)	C19—C20—H20	119.8
N2—C12—C21	116.57 (10)	C15—C20—H20	119.8
C12—C13—C14	124.36 (10)	C12—C21—H21A	109.5
O1—C14—N3	123.42 (11)	C12—C21—H21B	109.5
O1—C14—C13	122.24 (10)	H21A—C21—H21B	109.5
N3—C14—C13	114.33 (10)	C12—C21—H21C	109.5
C16—C15—C20	119.32 (11)	H21A—C21—H21C	109.5
C16—C15—N3	123.66 (10)	H21B—C21—H21C	109.5
C20—C15—N3	116.96 (10)	H99A—O99—H99B	109 (2)
			
C11A—C1—C2—C3	0.94 (19)	C4—C4A—C11A—C1	−0.60 (17)
C1—C2—C3—C4	−0.7 (2)	C4B—C4A—C11A—C1	179.61 (10)
C2—C3—C4—C4A	−0.3 (2)	C4—C4A—C11A—C11	178.48 (11)
C3—C4—C4A—C11A	0.89 (18)	C4B—C4A—C11A—C11	−1.31 (12)
C3—C4—C4A—C4B	−179.38 (12)	C11—N1—N2—C12	−176.41 (10)
C4—C4A—C4B—N5	3.0 (2)	N2—N1—C11—C11A	178.10 (10)
C11A—C4A—C4B—N5	−177.20 (11)	N2—N1—C11—C10A	−1.40 (18)
C4—C4A—C4B—C10A	−178.52 (12)	C1—C11A—C11—N1	0.22 (19)
C11A—C4A—C4B—C10A	1.24 (12)	C4A—C11A—C11—N1	−178.73 (10)
C10A—C4B—N5—C5A	1.18 (15)	C1—C11A—C11—C10A	179.83 (11)
C4A—C4B—N5—C5A	179.42 (10)	C4A—C11A—C11—C10A	0.87 (12)
C4B—N5—C5A—C6	179.66 (10)	N10—C10A—C11—N1	−1.8 (2)
C4B—N5—C5A—C9A	−0.54 (15)	C4B—C10A—C11—N1	179.47 (11)
N5—C5A—C6—C7	179.41 (11)	N10—C10A—C11—C11A	178.67 (11)
C9A—C5A—C6—C7	−0.39 (17)	C4B—C10A—C11—C11A	−0.09 (12)
C5A—C6—C7—C8	−0.19 (18)	N1—N2—C12—C13	−179.84 (10)
C6—C7—C8—C9	0.69 (18)	N1—N2—C12—C21	−0.30 (15)
C7—C8—C9—C9A	−0.59 (18)	N2—C12—C13—C14	−0.31 (18)
C8—C9—C9A—N10	−178.95 (10)	C21—C12—C13—C14	−179.83 (11)
C8—C9—C9A—C5A	0.00 (17)	C15—N3—C14—O1	−4.1 (2)
N5—C5A—C9A—N10	−0.39 (16)	C15—N3—C14—C13	176.42 (11)
C6—C5A—C9A—N10	179.41 (10)	C12—C13—C14—O1	−4.71 (19)
N5—C5A—C9A—C9	−179.31 (10)	C12—C13—C14—N3	174.77 (11)
C6—C5A—C9A—C9	0.49 (16)	C14—N3—C15—C16	−15.78 (19)
C9—C9A—N10—C10A	179.56 (10)	C14—N3—C15—C20	167.07 (12)
C5A—C9A—N10—C10A	0.64 (15)	C20—C15—C16—C17	0.02 (18)
C9A—N10—C10A—C4B	−0.02 (15)	N3—C15—C16—C17	−177.07 (11)
C9A—N10—C10A—C11	−178.59 (10)	C15—C16—C17—C18	−0.9 (2)
N5—C4B—C10A—N10	−0.97 (17)	C16—C17—C18—C19	0.9 (2)
C4A—C4B—C10A—N10	−179.52 (10)	C17—C18—C19—C20	0.1 (2)
N5—C4B—C10A—C11	177.85 (10)	C18—C19—C20—C15	−1.0 (2)
C4A—C4B—C10A—C11	−0.69 (12)	C16—C15—C20—C19	0.93 (19)
C2—C1—C11A—C4A	−0.33 (17)	N3—C15—C20—C19	178.21 (12)
C2—C1—C11A—C11	−179.19 (11)		

### (Z)-3-{2-[(Z)-11H-Indeno[1,2-b]quinoxalin-11-ylidene]hydrazinyl}-N-phenylbut-2-enamide monohydrate Hydrogen-bond geometry (Å, º)

**Table:**

*D*—H···*A*	*D*—H	H···*A*	*D*···*A*	*D*—H···*A*
N2—H02···N10	0.905 (18)	2.251 (18)	2.9178 (13)	130.2 (14)
N2—H02···O1	0.905 (18)	2.008 (17)	2.6860 (13)	130.5 (15)
N3—H03···O99^i^	0.916 (18)	1.920 (19)	2.8305 (13)	172.7 (17)
O99—H99*B*···N5^ii^	0.88 (2)	2.04 (2)	2.9032 (14)	168 (2)
O99—H99*A*···O1	0.89 (3)	1.91 (3)	2.7802 (14)	166 (2)
C16—H16···O1	0.95	2.32	2.9050 (15)	119

Symmetry codes: (i) −*x*+3/2, *y*−1/2, *z*; (ii) −*x*+1, −*y*+2, −*z*+1.
